# Microbial Biosurfactants in Cosmetic and Personal Skincare Pharmaceutical Formulations

**DOI:** 10.3390/pharmaceutics12111099

**Published:** 2020-11-16

**Authors:** Simms A. Adu, Patrick J. Naughton, Roger Marchant, Ibrahim M. Banat

**Affiliations:** 1 The Nutrition Innovation Centre for Food and Health (NICHE), School of Biomedical Sciences, Faculty of Life and Health Sciences, Ulster University, Coleraine BT52 1SA, Northern Ireland, UK; adu-s@ulster.ac.uk (S.A.A.); pj.naughton@ulster.ac.uk (P.J.N.); 2 Pharmaceutical Science Research Group, Biomedical Science Research Institute, Ulster University, Coleraine BT52 1SA, Northern Ireland, UK; r.marchant@ulster.ac.uk

**Keywords:** biosurfactants, cosmetics, glycolipids, lipopeptides, pharmaceutical formulations, skincare, surfactants

## Abstract

Cosmetic and personal care products are globally used and often applied directly on the human skin. According to a recent survey in Europe, the market value of cosmetic and personal care products in Western Europe reached about 84 billion euros in 2018 and are predicted to increase by approximately 6% by the end of 2020. With these significant sums of money spent annually on cosmetic and personal care products, along with chemical surfactants being the main ingredient in a number of their formulations, of which many have been reported to have the potential to cause detrimental effects such as allergic reactions and skin irritations to the human skin; hence, the need for the replacement of chemical surfactants with other compounds that would have less or no negative effects on skin health. Biosurfactants (surfactants of biological origin) have exhibited great potential such as lower toxicity, skin compatibility, protection and surface moisturizing effects which are key components for an effective skincare routine. This review discusses the antimicrobial, skin surface moisturizing and low toxicity properties of glycolipid and lipopeptide biosurfactants which could make them suitable substitutes for chemical surfactants in current cosmetic and personal skincare pharmaceutical formulations. Finally, we discuss some challenges and possible solutions for biosurfactant applications.

## 1. Introduction

The skin is a complex structure that constitutes the largest organ of the human body. Its primary function is to serve as a barrier, preventing excessive loss of moisture from the body; while on the outside, it prevents the entry of toxic substances and pathogens [1,2]. Histologically, the human skin is composed of three layers, namely, the epidermis, dermis and hypodermis [3]. Each of these layers contributes significantly to the functioning of the skin [4]. A complex network of interactions exists between epidermal cells and skin microbes, enabling the colonization of skin surfaces by a wide array of microorganisms, both commensal and mutualistic [5,6]. Different niches (moist, dry and sebaceous) of the skin surface selectively facilitate the growth of these diverse groups of microorganisms [7,8]. Many skin-inhabiting microbes offer great benefits to their host; while some help to activate the innate immune system, others produce antimicrobial substances (e.g., bacteriocins), which inhibit the growth of pathogens [7]. It is clear, therefore, that the maintenance of an effective skin microflora is a critical aspect of human health and has to be accommodated within the external challenges applied to the skin in the form of natural environmental contaminants and the optional application of personal care and cleansing products.

Cosmetic and personal care products are often formulated to provide nutrients and protection to the skin, its flora and associated cells, in addition to improving barrier functions, inhibiting the growth of pathogens, cleansing and moisturizing skin surfaces, all of which improve the skin’s overall health. In pursuit of the above, researchers and manufacturers of personal skincare products have been actively investigating new and promising ingredients to add to their formulations [9,10,11]. At present, many manufacturers of personal skincare products use chemical surfactants as ingredients in their formulations, particularly as emulsifiers and foaming agents. About 50% of chemical surfactants on the market are of petrochemical origin and are therefore derived from non-sustainable resources [12]. Although these chemical surfactants are extremely effective in formulations, it is suggested that they could be detrimental to the skin and its microbiome [13,14]. It is reported that some of these products often alter the skin flora, causing allergic reactions and skin irritations as they bind to lipids and proteins on the epidermal layer of the skin. Additionally, their prolonged use and high concentration may cause solubilization of the epidermal and intracellular lipids of the skin, thus, affecting the structural integrity and barrier functions of the skin [15]. For these reasons, there has been an impetus for the replacement of chemical surfactants with other compounds that can be produced from cheaper and sustainable resources and, additionally, have properties such as low toxicity, biodegradability and compatibility with the human skin, thereby having less or no negative effects on the health of consumers and the environment [13,16,17,18].

Biosurfactants are surface-active agents of biological origin, mainly produced by bacteria, yeast or filamentous fungi as secondary metabolites. Biosurfactants are obtained from these microorganisms through separation processes such as extraction, precipitation and distillation without adding any organic synthesis before, during and after production and as such, biosurfactants are also termed as naturally derived surfactants. Biosurfactants are generally neutral or anionic in nature. However, those that contain amine groups are cationic in nature [19,20,21]. The diverse structure of biosurfactants results from their different microbial origin, the substrate on which they are grown and cultivation conditions used [20]. Examples of biosurfactants that have been extensively studied include rhamnolipids, sophorolipids, mannosylerythritol lipids (MELs) and surfactin [22]. Biosurfactants have several potential advantages over their synthetic counterparts in addition to their wetting, emulsification, surface tension reduction and detergency functions. These potential advantages include lower toxicity, biodegradability, compatibility with the human skin, stability at extreme conditions (pH, temperature and salinity) and production from cheaper and renewable resources [17,21,23,24]. For these reasons, biosurfactants have received considerable attention in recent decades in the food, environmental protection, textile, oil, agriculture, cosmetic, medical and pharmaceutical industries [25].

Many reviews have focused on biosurfactant production, their characterization and application in the fields of environmental protection, oil refinery, food and agriculture. However, to our knowledge, there has been less attention paid to the potential application of microbial biosurfactants in the pharmaceutical, cosmetic and personal care industries. This review, therefore, focuses on the potential beneficial effects of biosurfactants on the human skin, its microbiome and associated cells, that could make them suitable to substitute for chemical surfactants in current cosmetic and personal skincare pharmaceutical formulations; specifically, their antimicrobial, skin moisturizing and low toxicity properties.

## 2. Classification of Biosurfactants

According to the current literature, biosurfactants are generally categorized into low and high molecular weight compounds. The low molecular weight biosurfactants are more effective at reducing surface and interfacial tensions whereas high molecular weight biosurfactants are better emulsifiers [19]. Each of these groups are further categorized into different classes based on their chemical composition [26,27]. The major classes of low molecular weight biosurfactants include glycolipids, lipopeptides, glycopeptides and phospholipids while high molecular weight biosurfactants include lipoproteins, polysaccharide-protein-fatty acid complexes and lipopolysaccharide-protein complexes [19,28,29]. However, for the purpose of this review, we will focus on low molecular weight biosurfactants, specifically glycolipids and lipopeptides.

Glycolipids constitute the most encountered and most promising group of biosurfactants in the cosmetic, pharmaceutical and food industries [30]. Glycolipids are composed of carbohydrate moieties bonded to fatty acid chains of varying lengths. Examples of glycolipids include rhamnolipids (mono- and di-rhamnolipids) of *Pseudomonas* spp., sophorolipids (Lactonic and acidic) and MELs (MELs A-C) of *Candida* spp., and trehalose lipids of *Mycobacterium* and *Rhodococcus* spp. [20,31,32].

Lipopeptides also have been in high demand in the therapeutic and biotechnological industries in recent years owing to their antimicrobial, antitumor and antitoxin potential [33]. Lipopeptides are mainly produced by *Bacillus* spp. The hydrophilic end of lipopeptides is generally composed of 7–10 amino acids and with hydrophobic component of fatty acids arranged in linear or cyclic order [34,35].

## 3. Biosurfactants as Promising Alternatives to Chemical Surfactants

Chemical surfactants are mainly classified based on the charge present on their hydrophilic heads after dissociation in water. They are therefore classified as cationic, anionic, amphoteric and non-ionic [36,37]. The amphiphilic structure and other unique properties of chemical surfactants allow their wide application in many current cosmetic and personal care products [15]. Examples of commercially available chemical surfactants mostly used in cosmetic and personal cleansing products include sodium dodecyl sulphate (SDS), sodium lauryl sulphate (SLS), cocamidopropyl betaine and cocamide diethanolamide [18,36]. These chemical surfactants are effective in carrying out the following functions: removal of skin and hair dirt; foaming to enhance lather in shampoos, emulsification or solubilization of immiscible liquids; skin and hair conditioning; moisturizing and wetting. Furthermore, they have absorption and surface tension reduction properties [15,36]. Despite these numerous benefits, it is reported that the prolonged use of cosmetic and personal care products formulated with chemical surfactants could have negative effects on the human skin. Paramount among these effects are alterations in the skin microbiome, skin irritations and allergic reactions, which may arise from the interaction of chemical surfactants with the epidermal layer of the skin [15,38].

The exact mechanisms underlying the detrimental effects that could be caused by chemical surfactants are not properly understood. However, it is believed that such drawbacks stem from the physical and chemical properties of chemical surfactants, the concentration used and duration of contact with the epidermis [18]. The epidermal layer of the skin is mainly composed of keratinocytes [39]. Keratinocytes undergo terminal differentiation to form corneocytes, which constitute the apical layer of epidermis called stratum corneum [40]. Corneocytes are embedded in a lipid-rich matrix and surrounded by a tough cross-linked cell envelope which confers their rigidity and protection [41]. Corneocytes are tightly connected with intracellular junctions called desmosomes. The intracellular spaces of corneocytes are filled with different types of lipids. These lipids are present in the following proportions: 50% ceramides, 25% free cholesterol, 10% cholesterol esters and 10% free fatty acids. These intracellular lipids together with hygroscopic compounds called natural moisturizing factors (NMFs) control transepidermal water loss (TEWL). The penetration of chemical surfactant unimers (individual monomer of surfactants) through the epidermis and its associated cells could cause a shift in balance of the different intracellular lipids (delipidation) and protein denaturation in the membranes of skin cells [42,43]. Furthermore, the interaction of some chemical surfactants with the skin can cause acute swelling of the stratum corneum which is often followed by deswelling [44]. Additionally, the penetration of chemical surfactants through the epidermis may affect living cells such as keratinocytes and Langerhans cells which form an integral part of the innate immune system, thereby affecting the overall immune responses [15].

The tendency of chemical surfactants to penetrate through skin layers causing protein denaturation, allergic reactions and skin irritations, among other factors, is largely dependent on the state of the surfactants in solution (i.e., monomer or micelle) and their concentration [44,45]. Micelles are formed from the aggregates of surfactant monomers in solution at a specific concentration called the critical micelle concentration (CMC) and temperature [46]. Some proposed theories on monomer, micelle and sub-micelle mechanisms of skin penetration remain debatable [47]. While some researchers claim that there is a reduction in rate of surfactant penetration through skin layers when the CMC is reached, owing to the relatively large size of micelles and their surface activities, others report that both micelles and monomers of chemical surfactants have the tendency to penetrate through skin layers and associated cells; and given that micelles are unstable, they may disintegrate into monomers after coming into contact with the skin. Additionally, micelles smaller than aqueous pore of stratum corneum could penetrate through the skin. Moreover, other small-sized micelles (sub-micelles) emerging during the continuous micelle formation and disintegration also have the potential to penetrate through the skin [18,47].

Some well-known approaches to address the effects of chemical surfactants on the human skin have been to increase the size of the surfactant hydrophilic component, the use of mixed surfactants (e.g., anionic and amphoteric surfactants) in formulations and ultimately, the use of surfactants with low CMC [44]. Moreover, in recent years, developments in the field of microbial biotechnology have expanded research into investigating the production of surfactants from natural sources, which will not only have the potential to overcome the above challenges, but in addition, improve skin health [18,22,25]. As such, a promising alternative has been the use of microbial biosurfactants [12,16]. The potential benefits of microbial biosurfactants to the human skin, its microbiome and associated cells will therefore be discussed in the next sections.

## 4. Biosurfactants, Human Skin and Its Microbiome

One square centimeter of the human skin has been estimated to contain about a billion microorganisms which include bacteria, fungi, virus and eukaryotic microorganisms [48]. Studies have shown that the human body is sterile before birth but becomes colonized by microbes during and after birth. Neonatal skin microbial diversity is dependent on the mode of birth, either by vaginal or assisted delivery (caesarean section). Vaginal delivery babies have a microbial community similar to that of their mother’s birth canal while those delivered by caesarean section have microbiota resembling the microbial community of their mother’s skin surfaces. Subsequent exposure to environmental microbes, mediated by a wave of activated immune system modulators called regulatory T lymphocytes (*T-reg* cells) and other host factors such as gender, location, nutrition and the use of cosmetic and personal care products often expand the skin’s microbial diversity [38,49,50].

The launching of the Human Microbiome Project (HMP) in 2008 in the United States of America (USA), and the use of advanced molecular biology approaches such as 16S rRNA and whole genome shotgun metagenomic sequencing, have revolutionized our understanding of the skin microbial community [49]. It has been further understood that bacteria are the predominant skin microbes at the kingdom level of microbial classification, having approximately equal interpersonal and intrapersonal variations [51]. Using 16S rRNA phenotyping, Grice et al. [52] detected 19 bacteria phyla from a study of 20 diverse skin sites of 10 healthy individuals. Most of the sequences were assigned to four phyla as follows: *Actinobacteria* (52%), *Firmicutes* (24%), *Proteobacteria* (17%) and *Bacteroidetes* (6%). They also found that *Cutibacterium* spp. and *Staphylococci* spp. were dominant in sebaceous areas and although *Staphylococci* spp. were present in moist areas, *Corynebacteria* spp. were the predominant. Moreover, dry areas of the skin such as volar forearm, hypothenar and the buttocks had mixed populations of bacterial cells [52]. More recently, Cosseau et al. [53] also detected the four major phyla abovementioned from the volar forearm of two healthy donors using both culture dependent (traditional method of bacterial identification) and culture independent (16S gene sequencing) methods of bacterial identification. Although Gram negative cells were present, they occupied a very small percentage (approximately 9.7%). Moreover, those Gram negative bacterial cells were mainly detected by gene sequencing [53]. The skin is an ecosystem on its own and it has a very responsive immune system modulating both pathogenic and commensal microbes [1,54]. Notwithstanding, the idea to incorporate bioactive compounds such as microbial biosurfactants into cosmetic and personal care products to encourage a balanced skin microbiome has long been postulated [13,21,31].

Biosurfactants have important potential physiochemical properties which are valuable for the maintenance of skin health. For instance, their fatty acid ends are effective for moisturizing rough and dry skin surfaces. Furthermore, *Cutibacterium acnes* (*C. acnes*) (formerly known as *Propionibacterium acnes*) hydrolysis of triglycerides in the fatty acid chain of microbial biosurfactants could help maintain the acidic pH of the skin, thereby encouraging the adherence of resident skin flora and discouraging the growth of pathogenic skin microbes to maintain a healthy skin microbiome. Additionally, the fatty acids could act as antioxidants to prevent the generation of free radicals by UV light [8,31,55,56].

Unlike chemical surfactants, the components of biosurfactants (sugars, lipids and proteins) are similar to those found in the membrane of skin cells (phospholipids and proteins). Moreover, the movement of compounds across the membrane of skin cells is dependent on their lipophilicity and surface activity, therefore, the unique structure of biosurfactants offers them a high rate of permeability through the membrane of skin cells to regulate protein and skin barrier functions, and trigger beneficial effects relating to hair repair and skin protection mechanisms [9,31,57]. Additionally, several in vitro studies have demonstrated that rhamnolipids, sophorolipids, MELs and surfactin are compatible with the human skin [22,31,58]. Furthermore, their emulsification, foaming, wetting and solubilizing functions, which are dependent on their chemical structure, make them desirable for use as ingredients in creams, lotions, powder, shampoos and other essential cosmetic products applied on the skin [59]. Current commercial cosmetic and skincare products containing microbial biosurfactants include Relipidium^TM^ (body and face moisturizer, produced by BASF, Monheim, Germany) [60], Sopholiance^TM^ S (deodorant, face cleaner and shower gel, produced by Givaudan Active Beauty, Paris, France) [61], Kanebo skincare (moisturizer, cleansing and UV filter, produced by Kanebo Cosmetics, Tokyo, Japan) [28], etc.

Natural inhibitory substances such as bacteriocins, enzymes, and alpha and beta defensins present on the skin surfaces help to keep its microbiome in constant check against pathogens [13,31]. In addition to the skin compatibility features of microbial biosurfactants, their potential to be effective in skin treatment therapies have been reported. Several biosurfactants have been demonstrated to have effective inhibitory mechanisms against skin pathogens such as *Pseudomonas aeruginosa* (*P. aeruginosa*), *Staphylococcus aureus* (*S. aureus*), *Streptococcus pyogenes* and *C. acnes.* For this reason, biosurfactants have been suggested for use as an alternative to conventional antibiotics, although the biocidal activity of biosurfactants is often slight and variable from one compound to another and their overall congener profile [59,62].

## 5. Antimicrobial Efficacy of Microbial Biosurfactants

Between 1930 and 1962, the world pharmaceutical industry produced more than 20 classes of new antibiotics [63]. This significant breakthrough in medicine drastically reduced the mortality rate of bacterial-associated infections. However, in the last 60 years, there has been a remarkable decline in their discovery and large-scale production to the market, such that only three new classes of antibiotics have been commercialized since then (i.e., mupirocin, oxazolidinone linezolid and lipopeptide daptomycin) [64]. Antibiotics are used in the treatment and prevention of a number of bacterial skin infections [65]. These infections include acne vulgaris, impetigo, eczema and atopic dermatitis (AD) [66,67,68]. Nonetheless, many bacteria that cause these infections have developed resistance to commonly used antibiotics due to their overuse; hence, the need for novel and more effective antimicrobial agents [65,69].

Biosurfactants that have antimicrobial properties have been reported in a number of studies [70,71,72]. Examples include rhamnolipids produced by *P. aeruginosa* [73], sophorolipids by *Starmerella bombicola* [22], mannosylerythritol lipids by genera *Ustilago* and *Pseudozyma*, surfactin produced by *Bacillus subtilis* (*B. subtilis*) [31,74], and others extracted from *Lactobacillus* spp. [75,76]. The antimicrobial activities of biosurfactants include antibacterial (bacteriostatic or bactericidal), antifungal, antiviral and antibiofilm effects [77,78].

The antimicrobial efficacy of biosurfactants is dependent on their structure, concentration used and class of bacteria under study [70,79]. Nashida et al. [80] used the chemical synthesis route to obtain 20 homologous members of MELs with different alkyl chain lengths and patterns of acyl groups and studied their antimicrobial effects on a wide range of bacterial cells including methicillin-resistant *Staphylococcus aureus* (MRSA), vancomycin resistant *Enterococci* (VRE), *Micrococcus luteus*, *Enterococcus faecalis* and *Enterococcus faecium* (VSE). The authors demonstrated that MEL-A with alkyl chain lengths of eight and ten carbons (C8 and C10) exhibited higher antimicrobial efficacy than those shorter (C6) and longer (C12 and C14) alkyl chain lengths, and that the antimicrobial efficacy of MELs was strongly influenced by the length of their alkyl chain. Although none of the synthesized compounds inhibited the growth of MRSA, MEL-D (C18) exhibited high antimicrobial effect on VSE and VRE, implying that the pattern of acyl groups on hydrophilic component of MELs is equally an important factor to consider, in addition to alky length, for antimicrobial efficacy of MELs [80]. Sophorolipids have also exhibited antimicrobial effects (bactericidal) against Gram positive pathogenic skin bacteria such as *S. aureus*, a major causative organism of AD, affecting 15–20% of children and 1–3% of adults worldwide, and *C. acnes*, which cause acne vulgaris in about 85% of teenagers [66,79]. The bactericidal effect of sophorolipids against these pathogenic skin bacteria has made them desirable for incorporation into personal skincare pharmaceutical formulations for the treatment of acne vulgaris, AD and body odor [21]. In addition to the concentration of sophorolipids used and class of bacteria investigated, the antimicrobial activity was dependent on the composition of their fatty acid chain, the sophorose group and congener used. For instance, although sophorolipids are reported to be generally less effective against Gram negative bacteria, their individual congeners have been shown to be more potent [79]. Lydon et al. [70] demonstrated the antimicrobial efficacy of acidic sophorolipids against Gram negative nosocomial infective agents such as *Escherichia coli* (*E. coli*) and *P. aeruginosa* at a concentration as low as 5 mg/mL. Meanwhile, it has been hypothesized that the synergy of sophorolipids with conventional antibiotics could increase the rate of antibiotics’ permeability through membrane of microbial cells, thereby achieving a higher antimicrobial effect [81]. In an attempt to validate this hypothesis, Juma et al. [71] used scanning electron microscopy (SEM) and atomic force microscopy (AFM) to investigate the mode of action and efficacy of sophorolipids and rhamnolipids when synergistically used with conventional antibiotics. They demonstrated that the synergistic use of sophorolipids with tetracycline, even at sub-minimum inhibition concentrations (sub-MICs), resulted in swelling and cell surface damage to MRSA, which was not observed for rhamnolipids [71].

Similar to sophorolipids, rhamnolipids are effective against a wide range of Gram positive bacteria but less effective against Gram negative bacteria [82]. Moreover, the antimicrobial efficacy of rhamnolipids against Gram positive bacteria has been reported to be pH dependent and is favored under acidic conditions [83]; thus, promising to be effective in skin treatment therapies, following other reports that the skin surface is mildly acidic (≈pH 5) [56,84,85]. De Freitas Ferreira et al. [83] demonstrated that the MIC of rhamnolipids against *S. aureus* at 39.1 μg/mL was bactericidal at pH 5.0 but bacteriostatic at pH 6.0. Surprisingly, at pH 7, the MIC was estimated to be above 250 μg/mL. It was further explained that at neutral or alkaline pH, rhamnolipids are anionic; however, at acidic pH, they act non-ionically, therefore, a stronger electrostatic force of attraction occurs between the non-ionic rhamnolipid carboxylic end and the anionic membrane of cells to achieve a higher antimicrobial effects [83].

Another valuable antimicrobial property of biosurfactants is their antibiofilm effects [86]. Biofilms are microbial complexes formed by one or more microorganisms on surfaces to enhance their survival, propagation and performance of other essential tasks [87]. Biofilm-associated skin infections include AD, acne vulgaris, chronic wounds and impetigo [88]. The relationship between biofilms and these skin infections have been demonstrated in several in vivo and in vitro studies, where the strong biofilm-producing potential of the causative organisms (i.e., *S. aureus*, *C. acnes, E. coli, P. aeruginosa,* etc.) was evident [88,89]. At biofilm state, these bacterial cells are embedded in a matrix composed of special substances called extracellular polymeric substances (EPS), consisting of polysaccharides, lipids, proteins, and extracellular DNA, and perform special physiological functions which often render them more virulent and resistant to antimicrobial agents, thereby exacerbating skin infections [90,91]. Nonetheless, in recent years, the efficacy of biosurfactants in the treatment of biofilms has been widely explored and they have been demonstrated to be promising for use in wound healing and skincare creams [25].

Several in vitro studies have demonstrated the efficacy of microbial biosurfactants in inhibiting the formation of new biofilms, preventing their adherence to surfaces [17,92] and the destruction of those already formed [93]. Karlapudi et al. [94] reported the inhibitory effect of glycolipid biosurfactants extracted from Acinetobacter M6 strain, achieving a reduction of 82.5% biofilm formation by the multi-drug resistant bacterium, MRSA, at a concentration of 500 µg/mL. Similarly, Rivardo et al. [92] reported a 97% biofilm inhibitory effect of the biosurfactant produced by *B. subtilis* V9T14 against *E. coli* CFT073. Moreover, rhamnolipid biosurfactants produced by *P. aeruginosa* in their biofilm development are critical for maintaining channels for the movement of fluids through biofilms by affecting cell–cell interaction and the attachment of bacterial cells on surfaces [95]. Additionally, rhamnolipids have the potential to induce biofilm detachments and dispersal, consequently rendering cells more susceptible to antimicrobial agents [96]. However, the pathogenicity status of *P. aeruginosa* producing rhamnolipids hinders their large-scale production and acceptance for use in food, cosmetic and pharmaceutical products because of the potential toxins present in them [97]. Nonetheless, currently, attention is being drawn to the production of biosurfactants from non-pathogenic microorganisms such as probiotic- and prebiotic-producing bacteria [98,99].

**Figure 1:**
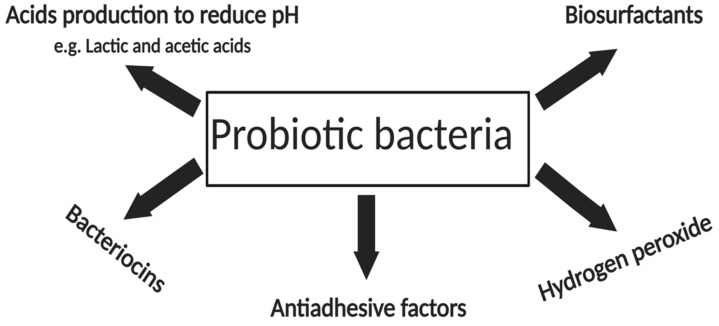
Antimicrobial activities of probiotic bacteria. Figure modified with permission from [102], John Wiley and Sons, 2016.

Probiotics are live microorganisms (probios) with health benefits [100,101] and are predominantly found in the gastrointestinal tract of humans and animals, and contribute significantly to preventing infections by inhibiting the growth of pathogenic microbes in the gut. The antimicrobial activities of probiotic organisms include the production of organic acids, bacteriocins, hydrogen peroxide, antiadhesion factors, and biosurfactant molecules, just to mention a few (Figure 1) [102,103,104]. An increasing number of studies have reported the production of biosurfactants from probiotic bacteria, which have been suggested to have additional potential benefits to human health aside from their antimicrobial effects, in that probiotic bacteria are innocuous to the normal human flora [105]. As such, biosurfactants produced from probiotic microorganisms could offer a substitute to biosurfactants such as rhamnolipids, mainly produced by pathogenic bacteria [106]. Among the diverse groups of probiotic biosurfactant-producing organisms, Lactic acid bacteria (LAB) are often predominant [99,106]. At present, information on the chemical composition of biosurfactants produced by LAB is quite limited [107]. However, the few structural analyses carried out suggest that biosurfactants produced by LAB may be composed of sugar, proteins, lipids or polysaccharide protein complexes associated with phosphate groups. The differences in the structural composition is dependent on the strain from which they are derived [99,108]. Although only a few *Lactobacillus* spp. are reported to produce glycolipid biosurfactants, they have been demonstrated to have high antimicrobial efficacy against a number of Gram positive and Gram negative multi-drug resistant pathogens in both planktonic and biofilm states in vitro [102].

Sharma and Singh [109] investigated glycolipid biosurfactants production by *Lactobacillus casei* MRTL3 using Fourier-transform infrared spectroscopy (FTIR). They reported the extracted compound contained carbohydrate and lipid moieties which was confirmed using 1H-Nuclear magnetic resonance. These biosurfactants demonstrated antibacterial effects against both *P. aeruginosa* and *S. aureus* [109]. Additionally, Sharma et al. [105] reported the preventative adhesion activity of glycolipid biosurfactants extracted from *Enterococcus faecium* MRTL9, demonstrating that biosurfactant concentrations as low as 25 mg/mL significantly reduced preformed biofilms in silicon tubes by *P. aeruginosa*, *E. coli* and *S. aureus*. Additionally, pre-adhesion antibiofilm assays on polystyrene surfaces revealed that the same concentration of the biosurfactants inhibited about 95% growth of biofilm for *E. coli* ATCC 25922, 89% for *P. aeruginosa* ATCC 15,442 and 83% for *S. aureus* ATCC 6538 [105]. More recently, Satpute et al. [76] investigated the antiadhesion and antibiofilm effects of biosurfactants derived from *Lactobacillus acidophilus*. Biosurfactant concentrations as low as 625 µg/mL inhibited the formation of *Proteus vulgaris* biofilms in commercial and medical grade catheter as well as inhibiting *B. subtilis* biofilm growth in microfluidic assemblies and on polydimethylsiloxane surfaces. These studies are noteworthy, given the effective antibacterial, antiadhesion and antibiofilm effects of biosurfactants extracted from probiotic organisms. Biosurfactants produced by probiotic bacteria may therefore have potential in the pharmaceutical and therapeutic industries as antimicrobial agents considering their non-pathogenic status and antimicrobial effects [99].

Prebiotics, on the other hand, are defined as indigestible food nutrients that selectively augment the growth and/or activities of beneficial gut microbes. The concept of prebiotics has been well established, particularly for probiotic organisms such as lactobacilli and bifidobacteria, which are predominant in the human colon [110]. Notwithstanding, in recent times, the potential role of prebiotics in maintaining a healthy skin microbiome is constantly being promoted by the cosmetic industries, although, so far, there are insufficient scientific data to back up this claim [54]. Currently, most commercially available antimicrobial agents do not only inhibit the growth of pathogenic microbes, but to some extent, affect the healthy skin microbiome. This often results in delayed healthy microbiota restoration and, for this reason, Schelges et al. [111] proposed, in a patent filed in 2016, the synergistic use of glycolipid biosurfactants (as prebiotics) with cosmetic cleansing agents, claiming to overcome the challenge of maintaining a balanced skin microbiome [111,112]. 

**Figure 2:**
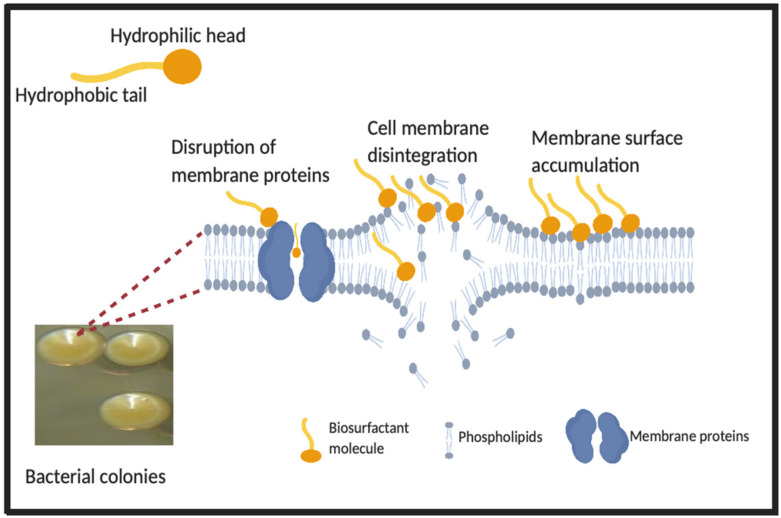
Theoretical interactions between biosurfactant molecules and bacterial cells. Primary mechanisms; disruption of cell membrane and proteins responsible for essential function. Figure adapted from [19,113] with permission from MDPI for [113], 2018 and created with BioRender.com.

Although microbial biosurfactants have been demonstrated to be effective antimicrobial agents against a number of pathogenic skin bacteria, their specific mechanisms of action are still not clear. It is speculated that the antimicrobial activities of biosurfactants are not dependent on a single mechanism. They may include intercalation between cellular phospholipid membranes, accumulation on membrane surfaces, membrane disintegration, removal of lipopolysaccharides or disruption of membrane proteins. However, primarily, they are believed to directly disrupt cell membranes or proteins responsible for essential membrane function as illustrated in Figure 2 [19,79,80,113]. For instance, the antimicrobial activities of surfactin biosurfactants are believed to be due to their accumulation on the bacterial cell membrane surfaces until a threshold concentration is reached, enabling their penetration into the membrane to cause further cellular disintegration [26]. Moreover, it is hypothesized that some biosurfactants insert their hydrophobic moieties into bacterial cell membrane in a flip-flop manner, creating pores, with the subsequent leakage of intracellular content as cells lose their integrity [78]. Indeed, most biosurfactants are more effective against Gram positive bacteria than Gram negatives [77]. It is suggested that the presence of the outer membrane structure in Gram negative bacteria offers them selectivity and extra protection [83]. Nevertheless, studies have shown that the majority of skin-inhabiting bacteria are Gram positives [1,8,114]. It has been explained that desiccation and high osmotic pressure on dry skin surfaces reduces the ability of Gram negative bacteria to colonize and multiply on such surfaces [115,116]. Therefore, the antimicrobial potential of microbial biosurfactants against pathogenic skin bacteria remains promising.

## 6. Biosurfactants as Skin Surface Moisturizer

Personal cleansing products such as shampoos are designed to be in contact with the skin and hair, but for a short while. Nevertheless, during this period, their interaction with the skin and its associated cells could affect the structural integrity of the stratum corneum, denature proteins, and solubilize intracellular lipids [47]. Microbial biosurfactants proposed as substitutes for chemical surfactants have been demonstrated to be compatible with the human skin and provide excellent skin surface moisturization [22,31,58,117]. The MELs produced by *Candida* spp. have particular application here. For instance, when the dried and damaged surface of an in vitro skin model, pre-treated with sodium dodecyl sulphate, was re-treated with 10% MEL-A glycolipid biosurfactants, cell viability increased to about 90% after 24 h of incubation [118]. Ceramide, an epidermal lipid, helps the formation of skin barrier and subsequently retains epidermal moisture. Studies have shown that the depletion of ceramides in the stratum corneum is a contributing factor to the etiology of skin diseases such as atopic dermatitis, eczema and psoriasis [119]. Natural or synthetic ceramides are good at improving skin surface roughness but are very expensive in production [120]. Therefore, MELs having similar properties offer a suitable replacement at a lower production cost [121]. MELs have also been reported to have moisturizing [122], water retention [123], rough skin and skin cells’ recovery effects [118].

**Figure 3:**
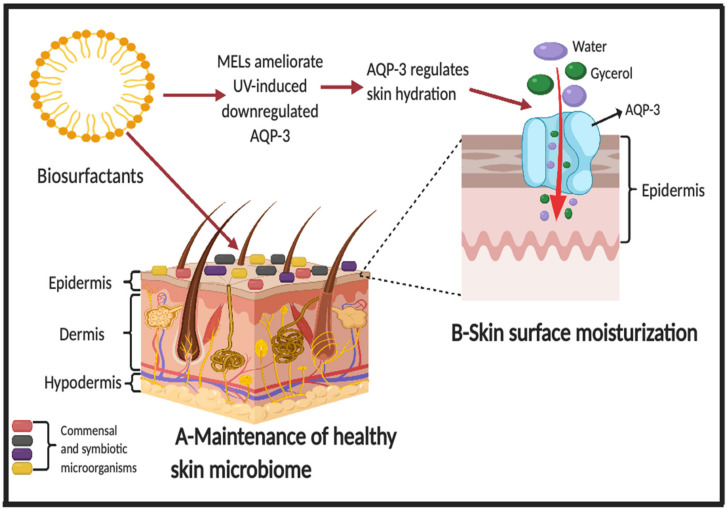
Potential benefits of microbial glycolipid and lipopeptide biosurfactants on human skin and its microbiome. (**A**) Maintenance of healthy skin microbiome; (**B**) Skin surface moisturization. AQP-3 = Aquaporin 3, MELs = Mannosylerythritol lipids. Figure adapted from [3,31,128] with permission from Taylor & Francis for [31], 2017 and created with BioRender.com.

Most often, the application of MELs in cosmetic products is related to their potential to increase water retention in the stratum corneum and to repair damaged hair [124]. Aquaporins (AQP) are a family of proteins that form water channels in the cell membrane of plants, bacteria and mammals. In mammals, there are 13 AQP (0–12). These membrane proteins allow the passage of water and other small solutes such as urea and glycerol across the skin epidermis, thus regulating various skin parameters such as hydration. AQP-3 is the most abundant and most studied aquaporin in the human skin. It transports uncharged solutes such as glycerol and urea in addition to water, ensuring water balance in the epidermis and transport of small solutes [125,126]. The evaluation of the relationship between age and disease associated skin dryness and AQP-3 expression has shown that the reduction in AQP-3 synthesis at protein and mRNA levels has an influence on skin dryness [125,127]. Recently, Bae et al. [128] reported that MEL-B (95% purity) has the potential to ameliorate UV-induced downregulated AQP-3 in human keratinocytes and to restore barrier functions of skin, indicating that MELs are promising for use as an ingredient in skin moisturizers to maintain a healthy skin microbiome as illustrated in Figure 3. Takahashi et al. [129] reported the antioxidant and protective effects of MEL-C against H_2_O_2_-induced oxidative stress in human skin fibroblasts. Results showed that MEL-C had a 50.3% scavenging activity at 10 mg/mL, demonstrating the best radical scavenging activity of all glycolipids [129]. Hyperpigmentation (e.g., freckles) is caused by overproduction of melanin [130]. However, the use of MELs as an ingredient in skin whitening formulations has been reported to be promising in suppressing melanocyte production and improving skin tone [131].

Sophorolipids also have the potential to trigger beneficial events relating to damaged hair repair and skin protection. Sophorolipids are commercially produced as humectant by Kao Co. Ltd., Japan, and are used in their products such as lipsticks, hair and skin moisturizers. Furthermore, sophorolipids are hypothesized to reduce subcutaneous overload of the skin by leptin synthesis stimulation through adipocyte. Similarly, research have shown that rhamnolipids are biocompatible and ideal for use in cosmetic and personal skincare pharmaceutical formulations [22,132].

There are claims that the lipid component of biosurfactants in moisturizers could help their deep penetration into the skin to stimulate collagen renewal and control other factors that cause destruction of skin structure [22]. However, more data are required to understand the relationship between biosurfactants, the epidermal layer of the skin and its components (i.e., corneocyte, keratinocytes and natural moisturizing factors) with regard to performance of these functions [133].

## 7. Cytotoxicity Studies

Challenges associated with the use of synthetic ingredients in cosmetic formulations include their tendency to cause allergic reactions [15]. An epidemiological survey in the U.K. revealed that about 23% of females and 14% of males typically experience some form of an adverse reaction to the cosmetic products they use in a year. It was further stated that approximately 10% of these adverse effects were allergic reactions [134]. A number of commonly used cosmetic and personal skincare products such as deodorants and antiperspirants contain aluminum-based compounds [135]. There are hypotheses that some of these compounds have the potential to cause allergic reactions and, in some cases, have been suggested as a contributing factor in Alzheimer’s disease, although, there are insufficient valid scientific evidences to support these hypotheses [136,137,138]. Bouslimani et al. [38], however, demonstrated that petroleum-based compounds in skincare products, such as polypropylene glycol in antiperspirant, may persist on the skin (with a half-life of about 1.9 weeks) and could significantly alter the metabolomic and microbial diversity of the skin. These potential defects, although they may be slight, could be resolved by the use of biosurfactants [31].

The low degree of toxicity (ability to induce permanent cell damage in skin cells) exhibited by microbial biosurfactants is fundamental for their acceptance for use in the pharmaceutical and cosmetic industries [18,20]. Stipcevic et al. [117] demonstrated that the degree of toxicity of microbial biosurfactants is dose dependent. Moreover, several researchers have reported that biosurfactants such as sophorolipids, rhamnolipids, MELs and surfactin are less toxic to mammalian cells compared to their chemical counterparts, therefore, confirming their safety for use. Cytotoxicity and skin irritancy studies of several biosurfactants have been carried out using both in vivo and in vitro models [23,82,139,140]. However, in recent decades, much emphasis has been placed on in vitro studies, which has led to the development of 3D skin models that have a well-layered cellular structure and barrier functions similar to human skin [141]. Moreover, there has been considerable success in enhancing the performance of these 3D skin models including an increase in shelf life and incorporation of skin cells such as fibroblasts, keratinocytes and melanocytes [141,142]. In addition to the 3D in vitro human skin models, researchers have developed in vitro porcine skin models which have percutaneous absorption potentials similar to the human skin, producing comparable permeabilization characteristics [57,143]. The development of in vitro skin models has provided an alternative to the use of in vivo human and animal skin models in laboratory experiments, thereby addressing ethical concerns raised by the use of in vivo models [144]. Despite the considerable successes in the further modification of these skin models, to appropriately substantiate and validate their efficacy in toxicity studies of biosurfactants, it would be worth testing them under rigorous environmental conditions (e.g., UV and airflow), when exposed to biosurfactants, and carrying out subsequent histological analysis where possible. Table 1 lists some examples of commercially available 3D in vitro skin models commonly used in laboratory experiments with their respective features and functions.

## 8. Effects of Biosurfactants on Skin Cell Types

*P. aeruginosa* are rarely found on skin of healthy individuals but are often present in burns and chronic wounds of patients. Epidermal keratinocytes secrete psoriasin, an antimicrobial protein (AMP) in response to expression of flagellin by *P. aeruginosa* [148]. Meyer-Hoffert et al. [149] demonstrated that rhamnolipids produced by *P. aeruginosa* also have the potential to facilitate the secretion of psoriasin without coming into direct contact with skin bacteria and responding cells; hence, preventing skin surface colonization by pathogens without altering the normal skin flora and immune cells [149]. Additionally, rhamnolipids have exhibited proliferative effects on epidermal keratinocytes. Stipcevic et al. [150] reported that in the presence of serum-containing medium, 50 µg/mL of rhamnolipids (di-RL BAC-3) stimulated proliferation of neonatal keratinocyte while inhibiting proliferation of fibroblastic cells under the same cultivation conditions and concentration. For practical applications, this effect would be necessary for the development of wound healing creams as inhibition of fibroblastic cell differentiation helps to avoid delay in wound healing whereas proliferation of keratinocytes aids the re-epithelization of wounds [150]. Sophorolipids synthesized by horse oil hydrolysis had no significant toxicity effect at up to 50 µg/mL against fibroblastic cell lines when cell viability was measured using the thiazolyl blue tetrazolium bromide (MTT) assay. Interestingly, at low concentrations (0.1 µg/mL), sophorolipids demonstrated a stimulatory effect on the fibroblastic cells [140]. Palmitoleic, linoleic and unsaturated fatty acids are the major compounds in horse oil, which are similar to those on skin. Using this technique, Maeng et al. [140] produced high-quality sophorolipids with the potential to improve skin health.

The in vivo potential toxicity of SPB1 lipopeptide biosurfactant towards mice was evaluated by Sahnoun et al. [23]. An LD_50_ value was set at 475 mg/kg. It was demonstrated that the mice monitored for 28 days did not show any signs of mortality or unusual change in behavior and locomotion upon daily intra-peritoneal injection with doses of 47.5 mg/kg or less. Additionally, no dermal reactions, such as irritations, were observed. Moreover, the SPB1 lipopeptide biosurfactant did not have any significant effects on their hematological and serum biochemical data [23]. Similarly, Surfactin C, lipopeptide produced by *B. subtilis* BC1212 was tested in vitro and in vivo on ICR mice to determine their genotoxicity. The mice studies did not show any maternal toxicity, fetotoxicity or teratogenicity when administered at a daily dose of 500 mg/kg [139].

Following a number of reports that marine yeasts have several unique promising features over terrestrial yeasts, Senthil Balan et al. [151] investigated the cytotoxicity effects of a glycolipid biosurfactant called trigalactomargarate (Cybersan), produced by *Cyberlindnera saturn* strain SBPN-27 against 3T3 embryonic fibroblastic cells. They demonstrated that at 200 μg/mL, the percentage of cell viability after 24 h was 97%. Moreover, at 400 μg/mL, 600 μg/mL, 800 μg/mL and 1000 μg/mL concentrations, the percentage cell viabilities were 92%, 85%, 79% and 70%, respectively. These concentrations of Cybersan were adequate to inhibit 100% growth of human clinical pathogens when antimicrobial efficacy of Cybersan was investigated. Moreover, the Cybersan biosurfactant was far less toxic than well-known glycolipid biosurfactants [151]. Indeed, on the whole, biosurfactants can be considered non-toxic compounds in comparison to their chemically-synthesized alternatives.

## 9. Challenges and Solutions for Biosurfactant Applications

Moisturizing, protection, cleansing and prevention are the essential requirements for effective cosmetic and personal care products [10]. It must be pointed out that biosurfactants will only be used as substitutes for chemical surfactants in cosmetic and personal care products if they are able to deliver equal or better performance in their formulations and have a market price that makes them attractive. However, large-scale production and limited structural variability of microbial biosurfactants (e.g., MELs) still remains a problem, although a few have been successfully commercialized (e.g., sophorolipids and rhamnolipids) [152,153]. Nevertheless, genomic sequencing, metabolic engineering and the use of microbial enzymes, are emerging alternatives to optimizing yield and structural variability of biosurfactants. Therefore, these areas should be closely examined and evaluated [74]. Additionally, biosurfactants produced by microorganisms are generally produced as a mixture of congeners rather than a single compound, and since different congeners have been shown to have different bioactivities, pure preparation of individual congeners needs to be investigated to accurately determine the efficacy of biosurfactants and their appropriate concentrations for use [154]. 

The problem of pathogenicity status of some Gram negative biosurfactant-producing strains, specifically, the commonly rhamnolipid-producing organism, *P. aeruginosa*, a Group two pathogen in the U.K., is seen by some as a disincentive to the commercial exploitation of rhamnolipids. This organism is an opportunistic pathogen and can cause serious infections in specific instances. Further investigation is required concerning their virulence factors and having specific measures in place to safeguard their mass production and acceptance for use in cosmetic formulations [155]. It is worth mentioning that a route to production of rhamnolipids through metabolic engineering of a non-pathogenic host organism has been achieved by Evonik Industries, Germany, which opened the door to economic and safe use of rhamnolipids in personal care products.

There is a considerable body of reviews suggesting the desirability of prebiotic and probiotic bacterial use in the cosmetic industries. At present, the former is readily incorporated into products whereas the latter is applied topically or added as a food supplement in beverages [156,157]. In the field of microbial biotechnology, biosurfactants have been extracted from a few probiotic- and prebiotic-producing organisms, and have proven to be effective for their respective purposes [76]. Given that probiotic- and prebiotic-producing organisms are innocuous to the normal human flora, they could be used as substitutes for biosurfactants produced by pathogenic microorganisms, thereby increasing the acceptance of microbial biosurfactants in food, cosmetic and personal care products [106]. Therefore, knowledge of the genetic composition and appropriate substrate and cultivation conditions for probiotic and prebiotic biosurfactant-producing organisms would be needed to exploit this new avenue.

## 10. Conclusions

The human skin is the largest organ of the body and in conjunction with the immune system, forms a responsive ecosystem [158]. Nonetheless, the skin, primarily performing a barrier function, is constantly exposed to several exogenous factors such as pathogens and toxins which often impair its overall function [3,159]. Cosmetic and personal care products are therefore formulated to provide nutrients and protection to the skin, improve barrier functions, inhibit the growth of pathogens, and moisturize skin surfaces [9,10]. In this review, we have discussed the promising features of microbial biosurfactants in relation to their potential to enhance cosmetic and personal care products to performing these functions, should they be used in their formulations. Although the mechanisms of action of biosurfactants on the human skin are not fully understood at present, advancements in future techniques and technology would undoubtedly help address these gaps as the fields of microbial biotechnology, pharmaceutical and cosmetic science critically investigate the relationship between biosurfactants and human skin.

## Figures and Tables

**Table 1:** Commercially available 3D in vitro skin models.

Skin Model	Features	Functions (Tests)	Longevity (Days)	Supplier	References
Labskin	Polymerized fibrin dermal equivalent, air exposed and well- differentiated epidermis	Studies interaction between skin and its microbiome, antimicrobial testing, etc.	10–14	Innoven Ltd.,York, England UK	[141,145]
Episkin^™^	Composed of collagen lattice with human fibroblast, surface overlay of human differential epidermis (type IV collagen)	Toxicological assessment of chemicals and products, skin corrosion, etc.	14	SkinEthic Labs., Lyon, France	[146]
EpiDermFT^™^	Full-thickness model with keratin 5, keratin 10 and involucrin, mitotically- and metabolically-active layers, dry surface	Wound healing, skin hydration, anti-aging	14	MatTek Life Sciences, Ashland, MA, USA	[147]
MelanoDerm^™^	Human-derived epidermal keratinocyte and melanocyte, highly differentiated epidermis	Skin pigmentation, lighting efficacy of cosmetic formulations	14	MatTek Life Sciences, Ashland, MA, USA	[147]
Phenion^™^ FT LONG-LIFE skin model	Human skin fibroblast and keratinocyte from single donor, full-thickness skin model (3 mm)	Toxicological assessment of chemicals and products, wound healing, skin permeability, drug delivery, etc.	50	Henkel AG CO., Dusseldorf, Germany	[142]

## References

[1] Grice E.A., Segre J.A. (2011). The skin microbiome. Nat. Rev. Microbiol..

[2] Yousef H., Alhajj M., Sharma S. (2020). Anatomy, Skin (Integument), Epidermis. StatPearls.

[3] Agarwal S., Krishnamurthy K. (2020). Histology, Skin. StatPearls.

[4] Lopez-Ojeda W., Pandey A., Alhajj M., Oakley A.M. (2020). Anatomy, Skin (Integument). StatPearls.

[5] Simmering R., Breves R. (2009). Pre- and probiotic cosmetics. Hautarzt.

[6] Bay L., Barnes C.J., Fritz B.G., Thorsen J., Restrup M.E.M., Rasmussen L., Sørensen J.K., Hesselvig A.B., Odgaard A., Hansen A.J. (2020). Universal dermal microbiome in human skin. MBio.

[7] Schommer N.N., Gallo R.L. (2013). Structure and function of the human skin microbiome. Trends Microbiol..

[8] Timm C.M., Loomis K., Stone W., Mehoke T., Brensinger B., Pellicore M., Staniczenko P.P.A., Charles C., Nayak S., Karig D.K. (2020). Isolation and characterization of diverse microbial representatives from the human skin microbiome. Microbiome.

[9] Heinrich K., Heinrich U., Tronnier H. (2014). Influence of different cosmetic formulations on the human skin barrier. Skin Pharmacol. Physiol..

[10] Rodan K., Fields K., Majewski G., Falla T. (2016). Skincare Bootcamp. Plast. Reconstr. Surg. Glob. Open.

[11] Gupta P.L., Rajput M., Oza T., Trivedi U., Sanghvi G. (2019). Eminence of microbial products in cosmetic industry. Nat. Prod. Bioprospect..

[12] Akbari S., Abdurahman N.H., Yunus R.M., Fayaz F., Alara O.R. (2018). Biosurfactants—A new frontier for social and environmental safety: A mini review. Biotechnol. Res. Innov..

[13] Holland K.T., Bojar R.A. (2002). Cosmetics What is Their Influence on the Skin Microflora?. J. Clin. Dermatol..

[14] Lee H.J., Jeong S.E., Lee S., Kim S., Han H., Jeon C.O. (2018). Effects of cosmetics on the skin microbiome of facial cheeks with different hydration levels. Microbiologyopen.

[15] Bujak T., Wasilewski T., Nizioł-Łukaszewska Z. (2015). Role of macromolecules in the safety of use of body wash cosmetics. Colloids Surf. B Biointerfaces.

[16] Marchant R., Banat I.M. (2012). Biosurfactants: A sustainable replacement for chemical surfactants?. Biotechnol. Lett..

[17] Banat I.M., De Rienzo M.A.D., Quinn G.A. (2014). Microbial biofilms: Biosurfactants as antibiofilm agents. Appl. Microbiol. Biotechnol..

[18] Seweryn A. (2018). Interactions between surfactants and the skin – Theory and practice. Adv. Colloid Interface Sci..

[19] Banat I.M., Franzetti A., Gandolfi I., Bestetti G., Martinotti M.G., Fracchia L., Smyth T.J., Marchant R. (2010). Microbial biosurfactants production, applications and future potential. Appl. Microbiol. Biotechnol..

[20] Santos D.K.F., Rufino R.D., Luna J.M., Santos V.A., Sarubbo L.A. (2016). Biosurfactants: Multifunctional biomolecules of the 21st century. Int. J. Mol. Sci..

[21] Varvaresou A., Iakovou K. (2015). Biosurfactants in cosmetics and biopharmaceuticals. Lett. Appl. Microbiol..

[22] Lourith N., Kanlayavattanakul M. (2009). Natural surfactants used in cosmetics: Glycolipids. Int. J. Cosmet. Sci..

[23] Sahnoun R., Mnif I., Fetoui H., Gdoura R., Chaabouni K., Makni-Ayadi F., Kallel C., Ellouze-Chaabouni S., Ghribi D. (2014). Evaluation of Bacillus subtilis SPB1 lipopeptide biosurfactant toxicity towards mice. Int. J. Pept. Res. Ther..

[24] Vijayakumar S., Saravanan V. (2015). Biosurfactants-types, sources and applications. Res. J. Microbiol..

[25] Naughton P.J., Marchant R., Naughton V., Banat I.M. (2019). Microbial biosurfactants: Current trends and applications in agricultural and biomedical industries. J. Appl. Microbiol..

[26] Ndlovu T., Rautenbach M., Vosloo J.A., Khan S., Khan W. (2017). Characterisation and antimicrobial activity of biosurfactant extracts produced by Bacillus amyloliquefaciens and Pseudomonas aeruginosa isolated from a wastewater treatment plant. AMB Express.

[27] Sandeep L. (2017). Biosurfactant: Pharmaceutical Perspective. J. Anal. Pharm. Res..

[28] Sil J., Dandapat P., Das S. (2015). Health Care Applications of Different Biosurfactants: Review. Int. J. Sci. Res..

[29] Kubicki S., Bollinger A., Katzke N., Jaeger K.E., Loeschcke A., Thies S. (2019). Marine biosurfactants: Biosynthesis, structural diversity and biotechnological applications. Mar. Drugs.

[30] Lukic M., Pantelic I., Savic S. (2016). An overview of novel surfactants for formulation of cosmetics with certain emphasis on acidic active substances. Tenside Surfactants Deterg..

[31] Vecino X., Cruz J.M., Moldes A.B., Rodrigues L.R. (2017). Biosurfactants in cosmetic formulations: Trends and challenges. Crit. Rev. Biotechnol..

[32] Peyrat L., Tsafantakis N., Georgousaki K., Ouazzani J., Genilloud O., Trougakos I.P., Fokialakis N. (2019). Terrestrial Microorganisms: Cell Factories of Bioactive Molecules with Skin Protecting Applications. Molecules.

[33] Patel S., Ahmed S., Eswari J.S. (2015). Therapeutic cyclic lipopeptides mining from microbes: Latest strides and hurdles. World J. Microbiol. Biotechnol..

[34] Kanlayavattanakul M., Lourith N. (2010). Lipopeptides in cosmetics. Int. J. Cosmet. Sci..

[35] Meena K., Sharma A., Kanwar S. (2017). Lipopeptides: A Distinct Class of Antibiotics with Diverse Applications. Adv. Biotechnol. Microbiol..

[36] Corazza M., Lauriola M., Zappaterra M., Bianchi A., Virgili A. (2010). Surfactants, skin cleansing protagonists. J. Eur. Acad. Dermatol. Venereol..

[37] Falk N.A. (2019). Surfactants as Antimicrobials: A Brief Overview of Microbial Interfacial Chemistry and Surfactant Antimicrobial Activity. J. Surfactants Deterg..

[38] Bouslimani A., da Silva R., Kosciolek T., Janssen S., Callewaert C., Amir A., Dorrestein K., Melnik A.V., Zaramela L.S., Kim J.-N. (2019). The impact of skin care products on skin chemistry and microbiome dynamics. BMC Biol..

[39] Yanase K., Hatta I. (2018). Disruption of human stratum corneum lipid structure by sodium dodecyl sulphate. Int. J. Cosmet. Sci..

[40] Eckhart L., Tschachler E. (2018). Control of cell death-associated danger signals during cornification prevents autoinflammation of the skin. Exp. Dermatol..

[41] Dykes P. (1998). Surfactants and the skin. Int. J. Cosmet. Sci..

[42] Moore D.J., Rawlings A.V. (2017). The chemistry, function and (patho)physiology of stratum corneum barrier ceramides. Int. J. Cosmet. Sci..

[43] Spada F., Barnes T.M., Greive K.A. (2018). Skin hydration is significantly increased by a cream formulated to mimic the skin’s own natural moisturizing systems. Clin. Cosmet. Investig. Dermatol..

[44] Ananthapadmanabhan K.P., Moore D.J., Subramanyan K., Misra M., Meyer F. (2004). Cleansing without compromise: The impact of cleansers on the skin barrier and the technology of mild cleansing. Dermatol. Ther..

[45] Som I., Bhatia K., Yasir M. (2012). Status of surfactants as penetration enhancers in transdermal drug delivery. J. Pharm. Bioallied Sci..

[46] Tadros T., Tadros T. (2013). Critical Micelle Concentration. Encyclopedia of Colloid and Interface Science.

[47] Morris S.A.V., Thompson R.T., Glenn R.W., Ananthapadmanabhan K.P., Kasting G.B. (2019). Mechanisms of anionic surfactant penetration into human skin: Investigating monomer, micelle and submicellar aggregate penetration theories. Int. J. Cosmet. Sci..

[48] Cooper A.J., Weyrich L.S., Dixit S., Farrer A.G. (2015). The skin microbiome: Associations between altered microbial communities and disease. Australas. J. Dermatol..

[49] Cundell A.M. (2018). Microbial Ecology of the Human Skin. Microb. Ecol..

[50] Ohue Y., Nishikawa H. (2019). Regulatory T (Treg) cells in cancer: Can Treg cells be a new therapeutic target?. Cancer Sci..

[51] Lunjani N., Hlela C., O’Mahony L. (2019). Microbiome and skin biology. Curr. Opin. Allergy Clin. Immunol..

[52] Grice E.A., Kong H.H., Conlan S., Deming C.B., Davis J., Young A.C., Bouffard G.G., Blakesley R.W., Murray P.R., Green E.D. (2009). Topographical and temporal diversity of the human skin microbiome. Science.

[53] Cosseau C., Romano-Bertrand S., Duplan H., Lucas O., Ingrassia I., Pigasse C., Roques C., Jumas-Bilak E. (2016). Proteobacteria from the human skin microbiota: Species-level diversity and hypotheses. ONEHLT.

[54] Musthaq S., Mazuy A., Jakus J. (2018). The microbiome in dermatology. Clin. Dermatol..

[55] Rocha M.A., Bagatin E. (2018). Skin barrier and microbiome in acne. Arch. Dermatol. Res..

[56] Smith R., Russo J., Fiegel J., Brogden N. (2020). Antibiotic delivery strategies to treat skin infections when innate antimicrobial defense fails. Antibiotics.

[57] Alonso C., Lucas R., Barba C., Marti M., Rubio L., Comelles F., Morales J.C., Coderch L., Parra J.L. (2015). Skin delivery of antioxidant surfactants based on gallic acid and hydroxytyrosol. J. Pharm. Pharmacol..

[58] Sen S., Borah S.N., Kandimalla R., Bora A., Deka S. (2020). Sophorolipid Biosurfactant Can Control Cutaneous Dermatophytosis Caused by Trichophyton mentagrophytes. Front. Microbiol..

[59] Gharaei-Fathabad E. (2011). Biosurfactants in pharmaceutical industry: A mini-review. Am. J. Drug Discov. Dev..

[60] BASF’s care creations. https://www.carecreations.basf.com/product-formulations/products/products-detail/RELIPIDIUMBC10096/307139450.

[61] Sopholiance^TM^ S. https://www.givaudan.com/fragrances/active-beauty/products/sopholiance-s.

[62] Findley K., Grice E.A. (2014). The Skin Microbiome: A Focus on Pathogens and Their Association with Skin Disease. PLoS Pathog..

[63] Yang Y., Ashworth A.J., Willett C., Cook K., Upadhyay A., Owens P.R., Ricke S.C., DeBruyn J.M., Moore P.A. (2019). Review of Antibiotic Resistance, Ecology, Dissemination, and Mitigation in U.S. Broiler Poultry Systems. Front. Microbiol..

[64] Butler M.S., Buss A.D. (2006). Natural products—The future scaffolds for novel antibiotics?. Biochem. Pharmacol..

[65] Lim J.S., Park H.S., Cho S., Yoon H.S. (2018). Antibiotic susceptibility and treatment response in bacterial skin infection. Ann. Dermatol..

[66] Sohn E. (2018). Skin microbiota’s community effort. Nature.

[67] Dempster C., Banat I., Mrrchant R. (2019). Antimicrobial and antibiofilm potential of biosurfactants as novel combination therapy against bacterium that cause skin infections. Access Microbiol..

[68] Oon H.H., Wong S.N., Wee D.C.A.W., Cheong W.K., Goh C.L., Tan H.H. (2019). Acne management guidelines by the Dermatological society of Singapore. J. Clin. Aesthet. Dermatol..

[69] Gudiña E., Teixeira J., Rodrigues L. (2016). Biosurfactants Produced by Marine Microorganisms with Therapeutic Applications. Mar. Drugs.

[70] Lydon H.L., Baccile N., Callaghan B., Marchant R., Mitchell C.A., Banat I.M. (2017). Adjuvant antibiotic activity of acidic sophorolipids with potential for facilitating wound healing. Antimicrob. Agents Chemother..

[71] Juma A., Lemoine P., Simpson A.B.J., Murray J., O’Hagan B.M.G., Naughton P.J., Dooley J.G., Banat I.M. (2020). Microscopic Investigation of the Combined Use of Antibiotics and Biosurfactants on Methicillin Resistant Staphylococcus aureus. Front. Microbiol..

[72] Anestopoulos I., Kiousi D.E., Klavaris A., Galanis A., Salek K., Euston S.R., Pappa A., Panayiotidis M.I. (2020). Surface active agents and their health-promoting properties: Molecules of multifunctional significance. Pharmaceutics.

[73] Zhao F., Shi R., Ma F., Han S., Zhang Y. (2018). Oxygen effects on rhamnolipids production by Pseudomonas aeruginosa. Microb. Cell Fact..

[74] Saika A., Koike H., Fukuoka T., Morita T. (2018). Tailor-made mannosylerythritol lipids: Current state and perspectives. Appl. Microbiol. Biotechnol..

[75] Sharma D., Saharan B.S., Chauhan N., Bansal A., Procha S. (2014). Production and Structural Characterization of Lactobacillus helveticus Derived Biosurfactant. Hindawi Publ. Corp..

[76] Satpute S.K., Mone N.S., Das P., Banpurkar A.G., Banat I.M. (2018). Lactobacillus acidophilus derived biosurfactant as a biofilm inhibitor: A promising investigation using microfluidic approach. Appl. Sci..

[77] Fernandes P.A.V., De Arruda I.R., Dos Santos A.F.A.B., De Araújo A.A., Maior A.M.S., Ximenes E.A. (2007). Antimicrobial activity of surfactants produced by Bacillus subtilis R14 against multidrug-resistant bacteria. Braz. J. Microbiol..

[78] Gomaa E.Z. (2013). Antimicrobial activity of a biosurfactant produced by bacillus licheniformis strain m104 grown on whey. Braz. Arch. Biol. Technol..

[79] Urzedo C.A., Freitas Q., Akemi V., Silveira I., Pedrine M.A., Celligoi C. (2018). Adv Biotech & Micro Antimicrobial Applications of Sophorolipid from Candida bombicola: A Promising Alternative to Conventional Drugs. Adv. Biotechnol. Microbiol..

[80] Nashida J., Nishi N., Takahashi Y., Hayashi C., Igarashi M., Takahashi D., Toshima K. (2018). Systematic and Stereoselective Total Synthesis of Mannosylerythritol Lipids and Evaluation of Their Antibacterial Activity. J. Org. Chem..

[81] Borsanyiova M., Patil A., Mukherji R., Prabhune A., Bopegamage S. (2016). Biological activity of sophorolipids and their possible use as antiviral agents. Folia Microbiol. (Praha).

[82] Bharali P., Das S., Ray A., Pradeep Singh S., Bora U., Kumar Konwar B., Singh C.B., Sahoo D. (2018). Biocompatibility natural effect of rhamnolipids in bioremediation process on different biological systems at the site of contamination. Bioremediat. J..

[83] De Freitas Ferreira J., Vieira E.A., Nitschke M. (2019). The antibacterial activity of rhamnolipid biosurfactant is pH dependent. Food Res. Int..

[84] Sekhon Randhawa K.K., Rahman P.K.S.M. (2014). Rhamnolipid biosurfactants- past, present, and future scenario of global market. Front. Microbiol..

[85] Nian Tan Y., Li Q. (2018). Microbial production of rhamnolipids using sugars as carbon sources. Microb. Cell Fact..

[86] Aleksic I., Petkovic M., Jovanovic M., Milivojevic D., Vasiljevic B., Nikodinovic-Runic J., Senerovic L. (2017). Anti-biofilm properties of bacterial di-rhamnolipids and their semi-synthetic amide derivatives. Front. Microbiol..

[87] López D., Vlamakis H., Kolter R. (2010). Biofilms. Cold Spring Harb. Perspect. Biol..

[88] Vaishnavi K.V., Safar L., Devi K. (2019). Biofilm in dermatology. J. Ski. Sex. Transm. Dis..

[89] Sonesson A., Przybyszewska K., Eriksson S., Mörgelin M., Kjellström S., Davies J., Potempa J., Schmidtchen A. (2017). Identification of bacterial biofilm and the Staphylococcus aureus derived protease, staphopain, on the skin surface of patients with atopic dermatitis. Sci. Rep..

[90] Flemming H.C., Neu T.R., Wozniak D.J. (2007). The EPS matrix: The “House of Biofilm Cells”. J. Bacteriol..

[91] Brandwein M., Steinberg D., Meshner S. (2016). Microbial biofilms and the human skin microbiome. NPJ Biofilms Microbiomes.

[92] Rivardo F., Turner R.J., Allegrone G., Ceri H., Martinotti M.G. (2009). Anti-adhesion activity of two biosurfactants produced by Bacillus spp. prevents biofilm formation of human bacterial pathogens. Appl. Microbiol. Biotechnol..

[93] Elshikh M., Moya-Ramírez I., Moens H., Roelants S., Soetaert W., Marchant R., Banat I.M. (2017). Rhamnolipids and lactonic sophorolipids: Natural antimicrobial surfactants for oral hygiene. J. Appl. Microbiol..

[94] Karlapudi A.P., Venkateswarulu T.C., Srirama K., Kota R.K., Mikkili I., Kodali V.P. (2020). Evaluation of anti-cancer, anti-microbial and anti-biofilm potential of biosurfactant extracted from an Acinetobacter M6 strain. J. King Saud Univ. Sci..

[95] Davey M.E., Caiazza N.C., O’Toole G.A. (2003). Rhamnolipid surfactant production affects biofilm architecture in Pseudomonas aeruginosa PAO1. J. Bacteriol..

[96] Kim S.K., Lee J.H. (2016). Biofilm dispersion in Pseudomonas aeruginosa. J. Microbiol..

[97] Uzoigwe C., Ennis C.J., Rahman P.K.S.M. (2015). Production of biosurfactants using eco-friendly microorganisms. Environmental Sustainability: Role of Green Technologies.

[98] Sharma D., Saharan B.S., Kapil S. (2016). Biosurfactants of Probiotic Lactic Acid Bacteria.

[99] Ghasemi A., Moosavi-Nasab M., Setoodeh P., Mesbahi G., Yousefi G. (2019). Biosurfactant Production by Lactic Acid Bacterium Pediococcus dextrinicus SHU1593 Grown on Different Carbon Sources: Strain Screening Followed by Product Characterization. Sci. Rep..

[100] Fijan S. (2014). Microorganisms with claimed probiotic properties: An overview of recent literature. Int. J. Environ. Res. Public Health.

[101] Gasbarrini G., Bonvicini F., Gramenzi A. (2016). Probiotics History. J. Clin. Gastroenterol..

[102] Satpute S.K., Kulkarni G.R., Banpurkar A.G., Banat I.M., Mone N.S., Patil R.H., Cameotra S.S. (2016). Biosurfactant/s from Lactobacilli species: Properties, challenges and potential biomedical applications. J. Basic Microbiol..

[103] Chen C.C., Lai C.C., Huang H.L., Huang W.Y., Toh H.S., Weng T.C., Chuang Y.C., Lu Y.C., Tang H.J. (2019). Antimicrobial activity of lactobacillus species against carbapenem-resistant enterobacteriaceae. Front. Microbiol..

[104] Barzegari A., Kheyrolahzadeh K., Mahdi S., Khatibi H., Sharifi S., Memar M.Y., Vahed S.Z. (2020). The battle of probiotics and their derivatives against biofilms. Infect. Drug Resist..

[105] Sharma D., Singh Saharan B., Chauhan N., Procha S., Lal S. (2015). Isolation and functional characterization of novel biosurfactant produced by Enterococcus faecium. Springerplus.

[106] Fariq A., Saeed A. (2016). Production and Biomedical Applications of Probiotic Biosurfactants. Curr. Microbiol..

[107] Morais I.M.C., Cordeiro A.L., Teixeira G.S., Domingues V.S., Nardi R.M.D., Monteiro A.S., Alves R.J., Siqueira E.P., Santos V.L. (2017). Biological and physicochemical properties of biosurfactants produced by Lactobacillus jensenii P 6A and Lactobacillus gasseri P 65. Microb. Cell Fact..

[108] Satpute S.K., Mone N.S., Das P., Banat I.M., Banpurkar A.G. (2019). Inhibition of pathogenic bacterial biofilms on PDMS based implants by L. acidophilus derived biosurfactant. BMC Microbiol..

[109] Sharma D., Singh Saharan B. (2014). Simultaneous production of biosurfactants and bacteriocins by probiotic lactobacillus casei MRTL3. Int. J. Microbiol..

[110] Davani-Davari D., Negahdaripour M., Karimzadeh I., Seifan M., Mohkam M., Masoumi S.J., Berenjian A., Ghasemi Y. (2019). Prebiotics: Definition, types, sources, mechanisms, and clinical applications. Foods.

[111] Schelges H., Tretyakova M., Ludwig B. Cleansing agents containing biosurfactants and having prebiotic activity. http://www.freepatentsonline.com/y2017/0071842.html.

[112] Reid G., Younes J.A., Van der Mei H.C., Gloor G.B., Knight R., Busscher H.J. (2010). Microbiota restoration: Natural and supplemented recovery of human microbial communities. Nat. Publ. Gr..

[113] Kaczorek E., Pacholak A., Zdarta A., Smułek W. (2018). The Impact of Biosurfactants on Microbial Cell Properties Leading to Hydrocarbon Bioavailability Increase. Colloids Interfaces.

[114] Cogen A.L., Nizet V., Gallo R.L., Richard Gallo C.L. (2008). Skin microbiota: A source of disease or defence? NIH Public Access. Br. J. Dermatol..

[115] Davis C.P., Baron S. (1996). Normal Flora. Medical Microbiology.

[116] Tarale P., Gawande S., Jambhulkar V. (2015). Antibiotic susceptibility profile of bacilli isolated from the skin of healthy humans. Braz. J. Microbiol..

[117] Stipcevic T., Piljac T., Isseroff R.R. (2005). Di-rhamnolipid from Pseudomonas aeruginosa displays differential effects on human keratinocyte and fibroblast cultures. J. Dermatol. Sci..

[118] Morita T., Kitagawa M., Suzuki M., Yamamoto S., Sogabe A., Yanagidani S., Imura T., Fukuoka T., Kitamoto D. (2009). A yeast glycolipid biosurfactant, mannosylerythritol lipid, shows potential moisturizing activity toward cultured human skin cells: The recovery effect of MEL-a on the SDS-damaged human skin cells. J. Oleo Sci..

[119] Choi M.J., Maibach H.I. (2005). Role of ceramides in barrier function of healthy and diseased skin. Am. J. Clin. Dermatol..

[120] Sethi A., Kaur T., Malhotra S.K., Gambhir M.L. (2016). Moisturizers: The slippery road. Indian J. Dermatol..

[121] Kitagawa M., Suzuki M., Yamamoto S., Sogabe A., Kitamoto D., Imura T., Fukuoka T., Morita T. Biosurfactant-containing skin care cosmetic and skin roughness-improving agent. http://www.freepatentsonline.com/y2010/0004472.html.

[122] Yamamoto S., Morita T., Fukuoka T., Imura T., Yanagidani S., Sogabe A., Kitamoto D., Kitagawa M. (2012). The moisturizing effects of glycolipid biosurfactants, mannosylerythritol lipids, on human skin. J. Oleo Sci..

[123] Lin T.-C., Chen C.-Y., Wang T.-C., Chen Y.-S. (2011). Characterization of Biosurfactant from a Diesel-oil Degradation Bacterium and Application Potential in Beauty Care Products. ICBEE.

[124] Paulino B.N., Pessôa M.G., Mano M.C.R., Molina G., Neri-Numa I.A., Pastore G.M. (2016). Current status in biotechnological production and applications of glycolipid biosurfactants. Appl. Microbiol. Biotechnol..

[125] Bonté F. (2011). Skin moisturization mechanisms: New data. Ann. Pharm. Fr..

[126] Patel R., Kevin Heard L., Chen X., Bollag W.B. (2017). Aquaporins in the skin. Advances in Experimental Medicine and Biology.

[127] Ikarashi N., Kon R., Kaneko M., Mizukami N., Kusunoki Y., Sugiyama K. (2017). Relationship between aging-related skin dryness and aquaporins. Int. J. Mol. Sci..

[128] Bae I.H., Lee S.H., Oh S., Choi H., Marinho P.A., Yoo J.W., Ko J.Y., Lee E.S., Lee T.R., Lee C.S. (2019). Mannosylerythritol lipids ameliorate ultraviolet A-induced aquaporin-3 downregulation by suppressing c-Jun N-terminal kinase phosphorylation in cultured human keratinocytes. Korean J. Physiol. Pharmacol..

[129] Takahashi M., Morita T., Fukuoka T., Imura T., Kitamoto D. (2012). Glycolipid biosurfactants, mannosylerythritol lipids, show antioxidant and protective effects against H 2O 2-induced oxidative stress in cultured human skin fibroblasts. J. Oleo Sci..

[130] Pillaiyar T., Manickam M., Jung S.H. (2017). Recent development of signaling pathways inhibitors of melanogenesis. Cell. Signal..

[131] Yoo J.W., Hwang Y.K., Bin S., Kim Y.J., Lee J.H. Skin whitening composition containing mannosylerythritol lipid 2019. http://www.freepatentsonline.com/y2019/0231668.html.

[132] Irfan-Maqsood M., Seddiq-Shams M. (2014). Rhamnolipids: Well-Characterized Glycolipids with Potential Broad Applicability as Biosurfactants. Ind. Biotechnol..

[133] Murphrey M.B., Miao J.H., Zito P.M. (2020). Histology, Stratum Corneum. StatPearls.

[134] Orton D.I., Wilkinson J.D. (2004). Cosmetic allergy: Incidence, diagnosis, and management. Am. J. Clin. Dermatol..

[135] Zirwas M.J., Moennich J. (2008). Antiperspirant and deodorant allergy: Diagnosis and management. J. Clin. Aesthet. Dermatol..

[136] Yegambaram M., Manivannan B., Beach T., Halden R. (2015). Role of Environmental Contaminants in the Etiology of Alzheimer’s Disease: A Review. Curr. Alzheimer Res..

[137] Exley C. (2017). Aluminum Should Now Be Considered a Primary Etiological Factor in Alzheimer’s Disease. J. Alzheimer’s Dis. Rep..

[138] German Federal Institute for Risk Assessment (Bfr) (2020). New studies on antiperspirants containing aluminium: Impairments to health unlikely as a result of aluminium uptake via the skin; Berlin, Germany. https//www.bfr.bund.de.

[139] Hwang Y.-H., Park B.-K., Lim J.-H., Kim M.-S., Song I.-B., Park S.-C., Yun H.-I. (2008). Evaluation of Genetic and Developmental Toxicity of Surfactin C from Bacillus subtilis BC1212. J. Heal. Sci..

[140] Maeng Y., Kim K.T., Zhou X., Jin L., Kim K.S., Kim Y.H., Lee S., Park J.H., Chen X., Kong M. (2018). A novel microbial technique for producing high-quality sophorolipids from horse oil suitable for cosmetic applications. Microb. Biotechnol..

[141] Bojar R.A. (2015). Studying the Human Skin Microbiome Using 3D In Vitro Skin Models. Appl. Vitr. Toxicol..

[142] Henkel AG & CO Press release Another milestone reached in skin research. www.henkel.com/press.

[143] Wang Y., Tan X., Xi C., Phillips K.S. (2018). Removal of Staphylococcus aureus from skin using a combination antibiofilm approach. NPJ Biofilms Microbiomes.

[144] Neupane R., Boddu S.H.S., Renukuntla J., Babu R.J., Tiwari A.K. (2020). Alternatives to biological skin in permeation studies: Current trends and possibilities. Pharmaceutics.

[145] Duffy E., De Guzman K., Wallace R., Murphy R., Morrin A. (2017). cosmetics Non-Invasive Assessment of Skin Barrier Properties: Investigating Emerging Tools for In Vitro and In Vivo Applications. Cosmetics.

[146] De Wever B., Petersohn D., Mewes K.R. (2013). Overview of human three-dimensional (3D) skin models used for dermal toxicity assessment. Househ. Pers. Care Today.

[147] MatTek Life Sciences EpiDermFT in vitro 3D Tissue | MatTek Life Sciences. https://www.mattek.com/products/epidermft/.

[148] Abtin A., Eckhart L., Mildner M., Gruber F., Schröder J.-M., Tschachler E. (2008). Flagellin is the principal inducer of the antimicrobial peptide S100A7c (psoriasin) in human epidermal keratinocytes exposed to Escherichia coli. FASEB J..

[149] Meyer-Hoffert U., Zimmermann A., Czapp M., Bartels J., Koblyakova Y., Gläser R., Schröder J.M., Gerstel U. (2011). Flagellin delivery by Pseudomonas aeruginosa rhamnolipids induces the antimicrobial protein psoriasin in human skin. PLoS ONE.

[150] Stipcevic T., Piljac A., Piljac G. (2006). Enhanced healing of full-thickness burn wounds using di-rhamnolipid. Burns.

[151] Senthil Balan S., Ganesh Kumar C., Jayalakshmi S. (2019). Physicochemical, structural and biological evaluation of Cybersan (trigalactomargarate), a new glycolipid biosurfactant produced by a marine yeast, Cyberlindnera saturnus strain SBPN-27. Process Biochem..

[152] Perfumo A., Rancich I., Banat I.M. (2010). Possibilities and challenges for biosurfactants use in petroleum industry. Adv. Exp. Med. Biol..

[153] Ahmadi-Ashtiani H.R., Baldisserotto A., Cesa E., Manfredini S., Zadeh H.S., Gorab M.G., Khanahmadi M., Zakizadeh S., Buso P., Vertuani S. (2020). Microbial biosurfactants as key multifunctional ingredients for sustainable cosmetics. Cosmetics.

[154] Rahimi K., Lotfabad T.B., Jabeen F., Mohammad Ganji S. (2019). Cytotoxic effects of mono- and di-rhamnolipids from Pseudomonas aeruginosa MR01 on MCF-7 human breast cancer cells. Colloids Surf. B Biointerfaces.

[155] Marchant R., Banat I.M. (2012). Microbial biosurfactants: Challenges and opportunities for future exploitation. Trends Biotechnol..

[156] Farris P. Are Skincare Products with Probiotics Worth the Hype?. https://www.dermatologytimes.com/view/are-skincare-products-probiotics-worth-hype.

[157] Mantzourani I., Nouska C., Terpou A., Alexopoulos A., Bezirtzoglou E., Panayiotidis M.I., Galanis A., Plessas S. (2018). Production of a novel functional fruit beverage consisting of cornelian cherry juice and probiotic bacteria. Antioxidants.

[158] Matejuk A. (2018). Skin Immunity. Arch. Immunol. Ther. Exp. (Warsz).

[159] Prescott S.L., Larcombe D.-L., Logan A.C., West C., Burks W. (2017). The skin microbiome: Impact of modern environments on skin ecology, barrier integrity, and systemic immune programming. World Allergy Organ. J..

